# Pediatric Surgical Care During the COVID-19 Lockdown: What Has Changed and Future Perspectives for Restarting in Italy. The Point of View of the Italian Society of Pediatric Surgery

**DOI:** 10.3389/fped.2022.871819

**Published:** 2022-05-19

**Authors:** Francesco Morini, Carmelo Romeo, Fabio Chiarenza, Ciro Esposito, Piergiorgio Gamba, Fabrizio Gennari, Alessandro Inserra, Giovanni Cobellis, Ernesto Leva, Rossella Angotti, Alessandro Raffaele, Sebastiano Cacciaguerra, Mario Messina, Mario Lima, Gloria Pelizzo

**Affiliations:** ^1^Neonatal Surgery Unit, AOU Meyer and University of Florence, Florence, Italy; ^2^Department of Human Pathology of Adult and Childhood “Gaetano Barresi”, Unit of Pediatric Surgery, University of Messina, Messina, Italy; ^3^Division of Pediatric Surgery, San Bortolo Hospital, Vicenza, Italy; ^4^Division of Pediatric Surgery, Federico II University Hospital, Naples, Italy; ^5^Pediatric Surgery Unit, Women's and Children's Health Department, University Hospital of Padua, Padua, Italy; ^6^Department of Pediatric General Surgery, Regina Margherita Children's Hospital, Turin, Italy; ^7^Surgical Oncology-General and Thoracic Surgery Unit, Department of Surgery, Bambino Gesù Children Hospital IRCCS, Rome, Italy; ^8^Pediatric Surgery Unit, Salesi Children Hospital, Ancona, Italy; ^9^Department of Pediatric Surgery, Fondazione IRCCS Ca' Granda, Ospedale Maggiore Policlinico, Milan, Italy; ^10^Division of Pediatric Surgery, Department of Medical Sciences, Surgical Sciences and Neurosciences, Hospital of “Santa Maria Alle Scotte”, Siena, Italy; ^11^Department of Pediatric Surgery, Fondazione Istituti di Ricovero e Cura a Carattere Scientifico (IRCCS) Policlinico San Matteo, University of Pavia, Pavia, Italy; ^12^Department of Pediatric Surgery, Ospedale Garibaldi, Catania, Italy; ^13^Pediatric Surgery Unit, S. Orsola Hospital, University of Bologna, Bologna, Italy; ^14^Pediatric Surgery Department of Pediatric Surgery, “V. Buzzi” Children's Hospital, Milan, Italy; ^15^Department of Biomedical and Clinical Science “L. Sacco”, University of Milan, Milan, Italy

**Keywords:** child, COVID-19, criticalities, pediatric surgery, neonate

## Abstract

**Background:**

The coronavirus disease 2019 (COVID-19) time exacerbated some of the conditions already considered critical in pediatric health assistance before the pandemic. A new form of pediatric social abandonment has arisen leading to diagnostic delays in surgical disorders and a lack of support for the chronic ones. Health services were interrupted and ministerial appointments for pediatric surgical healthcare reprogramming were postponed. As a result, any determination to regulate the term “pediatric” specificity was lost. The aim is, while facing the critical issues exacerbated by the COVID-19 pandemic, to rebuild future perspectives of pediatric surgical care in Italy.

**Methods:**

Each Pediatric Society, including the Italian Society of Pediatric Surgery (SICP), was asked by the Italian Federation of Pediatric Associations and Scientific Societies to fill a questionnaire, including the following the main issues: evaluation of pre-pandemic criticalities, pediatric care during the pandemic and recovery, and current criticalities. The future care model of our specialty was analyzed in the second part of the questionnaire.

**Results:**

Children are seriously penalized both for surgical treatment as well as for the diagnostic component. In most centers, the pediatric surgical teams have been integrated with the adult ones and the specificity of training the pediatric operating nursing is in danger of survival. “Emotional” management of the child is not considered by the general management and the child has become again an adults patient of reduced size.

**Conclusion:**

A new functional pediatric surgical model needs to be established in general hospitals, including activities for day surgery and outpatient surgery. To support the care of the fragile child, a national health plan for the pediatric surgery is required.

## Introduction

The Italian Society of Pediatric Surgery (SICP) is one of the oldest in Europe, having been founded in Livorno on the 24 February 1963. It aims to promote the progress of surgical art and science in the pediatric field, to promote and maintain the highest standard in the quality of surgical treatment provided to children in Italy, to protect the prestige and interests of pediatric surgery and its practitioners, and to facilitate the relationships of association and the exchange of ideas between pediatric surgeons. Since those early days, pediatric surgery has enormously evolved and is today one of the most vigorously growing fields in surgery. Pediatric surgery is unique in many instances. It is one of the last true general surgeries. The pediatric surgeon deals with a completely distinctive set of disorders, namely, congenital malformations that are largely unknown to the general adult surgeon. Finally, the pediatric surgeon operates on a future, as most of patients have a long life expectancy in front of them. This brings with it the greatest responsibility for the caring surgeon. Despite, or maybe due to these peculiarities, pediatric surgery has long faced several critical issues. Diagnosis Related Groups (DRG) dedicated to the child are largely lacking, leading to the application of DRG created based on adult surgery necessities that do not take into account the peculiar aspects of the pediatric patient. Additionally, a clear definition of the pediatric patient is missing, which may change from region to region. As a consequence, pediatric patients may be treated in pediatric or general surgical units, depending on the region where they are located. This leads to treatment disparities due to the lack of specific pediatric expertise for patients admitted to general surgical units. In addition to the confusion on the definition of the pediatric patient, there are no projects involving the whole of Italy on the number of centers necessary to cover the needs of the population. The regions work without general coordination, which should give behavioral guidelines to the identification and authorization to open centers that should respond to clear and precise criteria. Finally, there is a shortage of pediatric surgeons in part due to mistakes made in the past regarding the forecast of future needs and in part to the fact that trainees in pediatric surgery have strict limitations on their clinical and surgical activity.

These critical issues were further exacerbated during the COVID-19 pandemic. Management of patients with COVID-19 became a priority. For all other patients, the pandemic modified and slowed down hospital admittance and disarrayed the daily routine programs of diagnosis and treatment. From a healthcare point of view, there was no determination to regulate the term “pediatric” specificity. Pediatricians and pediatric surgeons were moved to adult medicine, either directly to adult patients with COVID-19 or to “free” adult colleagues to be employed in COVID-19 units or hospitals (1, 2). The “stay at home” orders during the COVID-19 period have led to a complete change in the daily social activities of children. A new form of pediatric social abandonment has arisen with an increase in domestic accidents (3–5), obesity, and psychiatric disorders. There was above all a diagnostic delay in surgical disorders and a lack of support for the chronic ones (6–8).

As result, the COVID-19 time exacerbated some of the conditions already considered critical in pediatric health assistance before the pandemic. All elective health services were interrupted and ministerial appointments for pediatric healthcare reprogramming were postponed with the consequence that restrictions placed pediatric needs in the last position on the social ladder.

The aim of this report is, while facing the critical issues exacerbated by the COVID-19 pandemic, to rebuild future perspectives of pediatric surgical care in Italy. To propose a perspective of the surgical children's care, a collection of data from before and during the pandemic period, as well as the current situation, was considered.

## Methods

The Italian Society of Pediatric Surgery is institutionally included in the Italian Federation of Pediatric Associations and Scientific Societies (FIARPED). Each pediatric society was asked by the FIARPED to describe how their field was impacted by the COVID-19 pandemic following predefined questions (as shown in Appendix) as a canvas. The main issues of the questionnaire were: the evaluation of pre-pandemic criticalities, pediatric care during the pandemic and recovery, and current criticalities.

The future care model of our specialty was analyzed in the second part of the questionnaire.

## Results

Italy, with over 60 million inhabitants, has 66 pediatric surgery units. Among these, 22 units (33%) operate in exclusively pediatric hospitals, whereas, 21 units (31%) operate in institutions offering both adult and pediatric surgery services and belong to university centers.

### Pre-pandemic Criticalities

The type of pediatric care is completely different from that of adults with completely peculiar implications related to age, fragility, and complexity. Specific and updated DRGs are missing. The current ones are obsolete and most of the time derive from those of adults very far from the needs and complexity of pediatric ones. Although about 10 years ago, a special ministerial commission, including pediatricians and pediatric surgeons, studied and drafted a hypothesis, this hypothesis has never been supported as a project, nor applied to clinical practice.

The age of admittance in pediatric wards ranges between 0–14 and 0–18 years and they are strictly related to decisions established by local authorities or regions in almost all cases. As a consequence, the lack of shared indications creates confusion that finds its maximum expression in adolescent patients who are treated in both pediatric and adult centers, causing inequality in treatment and a lack of acquisition of correct expertise.

Moreover, there is a great deal of confusion between day surgery and outpatient surgery activities imposed by the regions, probably because they have been imported *sic and simpliciter* from adulthood without taking into account the objective limitations related to age and particular to the *fragile* pediatric patients. Not without greater importance, it should be emphasized that the uncontrolled proliferation of pediatric surgery units on the territory of the country due to a lack of national planning for the distribution. Currently, there are 56 pediatric surgery units in Italy and with about one million inhabitants per unit as a reference. The lack of a project involving the whole of Italy on the number of centers necessary to cover the needs of the population penalizes not only the existing ones but also the local new acquisitions. There is a formal and substantial disorder because the regions work without general coordination that should give behavioral guidelines to the identification and authorization to open new centers that should respond to clear and precise criteria. An adequate national plan with precise indications would facilitate the identification of existing references and real territorial needs.

It is not always when children are hospitalized that they end up in the pediatric wards. In Italy, about 25% of children do not receive care in child-friendly pediatric wards. The little ones are frequently and willingly enticed together with the older patients, potentially causing psychological discomforts for the child, who would need a more protective environment. According to the National Agency for Regional Health Services, Agenas, in 2019 over 175,000 hospitalizations of patients between 0 and 17 years, accounting for over 25% out of 695,215 admissions, were carried out in adult departments, in particular when surgery was required. The most frequent admissions in adult surgical units are for orthopedics, ENT, testis disorders, and appendicitis.

Last but not least, the problem of surgeons in training who, due to a reduction in number in the pediatric surgery area, do not help the necessary generational turnover. This point fits perfectly with the previous one. National central planning is needed to define the number of pediatric surgery units in the country.

### Pediatric Care During the Pandemic and Recovery

#### SICP at the Beginning of the Pandemic

To deal with the COVID-19 pandemic, SICP has identified the following recommendations to avoid and contain the infection, guaranteeing the protection of children, surgeons, anesthetists, nursing staff, and personnel belonging to the “surgical team,” providing timely surgical treatments to children with pathologies of surgical interest, and optimize the resources needed for child care. SICP identified a list of surgical emergencies (Tables 1, 2) for which a delay in treatment could represent a significant short-term threat to patient's life, therefore requiring immediate treatment.

**Table 1:** Surgical emergencies.

Acute intestinal occlusions
Volvulus
Incarcerated inguinal hernia
Hypertrophic pyloric stenosis
Acute intestinal intussusception (after contrast enema failure)
Need for extracorporeal life support (ECMO)
Intestinal perforation
Worsening necrotizing enterocolitis
Thoraco-abdominal trauma (closed, open and hemorrhagic)
Ischemia: testicular torsion, ovarian torsion, limb ischemia (iatrogenic or traumatic)
Congenital disorders:
- Esophageal atresia with T-E fistula
- Congenital and symptomatic diaphragmatic hernia
- Intestinal atresia
- Congenital intestinal occlusion (anorectal malformations; Hirschsprung's disease not responding to nursing)
Acute appendicitis with suspected peritonitis
Foreign body in the esophagus or trachea^*^
Burns requiring immediate treatment under sedation or general anesthesia

* *There is an increased risk for transmission of severe acute respiratory syndrome coronavirus 2 (SARS-CoV-2) in endoscopic procedures. Non-deferrable surgery. Additionally, a list of disorders that may be treated with some delay of few days to few weeks was highlighted by the Italian Society of Pediatric Surgery (SICP) (Table 2)*.

**Table 2:** Disorders treatable with deferred surgery.

Surgical oncology
Biliary atresia
Abscess incision and drainage
Inflammatory bowel disease not responsive to medical treatment
Central venous line insertion
Symptomatic inguinal hernia
Gallbladder surgery for symptomatic gallstones
Feeding gastrostomy (if needed for discharge)
Hydronephrosis with renal function impairment or with high risk for pyelonephritis
Surgery for urethral valves
Urethral stenosis

#### What Happened Compared to What We Had Assumed

The programming of the elective interventions has been taken, with everything that follows: instrumental examinations, anesthesiologic examinations, and expected date of intervention.

The pediatric surgery units in non-pediatric hub hospitals resulted in being seriously penalized concerning the scheduled activities that were expunged due to the wide use of the operating rooms as intensive care rooms for COVID-19 admittance. Every center suffered from a dramatic reduction of ward spaces because some rooms were dedicated to surgical patients with COVID-19, operating rooms in general hospitals were used for adults and children, and some pediatric hospitals were converted to intensive care units (ICUs). Some pediatric hospitals accepted adult patients with positive COVID-19 for a period in intensive care (9).

Pediatric surgeons needed to manage some patients with abdominal conditions that were different from those they were used to. Many pediatric centers were seriously affected by severe acute respiratory syndrome coronavirus 2 (SARS-CoV-2) related multisystem inflammatory syndrome in children (MIS-C), a new nosological entity that has mainly affected small children and adolescents with serious consequences (10, 11). In these patients, surgeons had to question whether or not a surgical approach was indicated, often *a posteriori*, when, entering an abdomen in the suspicion of acute appendicitis or peritonitis from complicated acute appendicitis, complex peritonitis in the absence of macroscopic appendiceal disease, or bowel ischemia without a macroscopically obvious explanation were found. The highest degree of abdominal involvement associated with lower age, even less than 5 years of age with a high risk of morbidity and mortality. The seriousness of this new disease has not been adequately taken into consideration by the national healthcare.

#### How Did We Reorganize Ourselves?

The centers have slowly and gradually rescheduled their activities, reclassifying the disorders according to recommendations given by the SICP. Reclassification of patients on the waiting list by severity classes was made, trying to highlight that even “routine” disorders (e.g., undescended testes) must be operated on at certain times (12).

### Current Criticalities (Table 3): What Are the Criticalities That Our Specialty Is Experiencing in This Moment of Change?

Due to the COVID-19 pandemic, the “emotional” aspects of the admitted child have lost their central position in patients' management. A single-parent admittance is allowed during the whole hospital stay, surveillance in the recovery room has completely disappeared, and the reception paths dedicated to fragile and chronic children have been suppressed. In addition, in non-pediatric hub centers (general hospitals), the child is heavily penalized and is not prioritized in emergencies compared with other adult surgical specialties. Children are seriously penalized both for surgical treatment as well as for the diagnostic component. In the operating theaters, the COVID-19 time brought together nurses and scrub nurses of various surgical specialties. The result is that surgical teams have been integrated with the adult ones and pediatric nurses and scrub nurses have been shifted to adult operating theaters. In this way, the specificity of training the pediatric nursing staff to manage the premature and critically low weight children is affected.

**Table 3:** Current criticalities in pediatric surgery units.

In general hospitals the surgical child has not priority in emergencies compared to other adult specialties, both for the surgical and diagnostic step
In the operating rooms, the need to place side by side nurses and scrub nurses of various specialties, to optimize the operating slots, led to loss of skills for the staff already trained for the pediatric patient
With the “excuse” of covid time the “emotional” containment of the child takes a back seat and the pediatric patient has become like an adult of “reduced size”
No paths designed to contain stress (single parent during hospital stay, disappearance of the recovery rooms)

The child has become again as an adult patient of smaller size.

## Discussion

The COVID-19 pandemic affected every phase of emergent and scheduled activity in pediatric surgery. Interactions with the territory, already critical in a pre-COVID-19 pandemic, indicated a lack of organization of the pediatric hub-spoke network.

The goal of pediatric surgeons is to ensure specific care dedicated to the pediatric patient, in a devoted environment, and with dedicated methods. The pediatric surgery unit must be the “container” of specialists in the subject and related specialties and must offer expertise in the care of the child. Infections of COVID-19 are reported to be milder in children than in the adult population. However, COVID-19 infection in children may cause concerns both for the risk of contributing to the spread of the infection and for the appearance of the MIS-C, which is considered a serious complication (13). To deal with the risk of spreading, several institutions have reorganized their pediatric departments to provide a separate flow for high-risk and low-risk patients with COVID-19 and increase the number of beds in dedicated negative pressure areas (14). This was also implemented in pediatric surgical departments. The categories at highest risk of MIS-C are children affected by disabilities, tumors, malformations associated with related syndromes, and patients with immunological deficiency. Based on lessons learned during the Covid-19 pandemic, the admission of these patients needs dedicated pediatric treatment paths. A better prognosis would be provided by a possible model of pediatric surgery reorganization capable of guaranteeing the respect of pediatric surgical patient flows during a pandemic event. The pediatric ICU of COVID-19 hospital should provide a temporary arrangement of the number of beds to obtain the recruitment of frailly sick children from the network between territorial pediatrics and hospitals.

A “functional pediatric surgical model” for the pediatric area can also be established within a general hospital (Table 4).

**Table 4:** Pediatric surgery future care model in general hospitals.

Give the child the whole dignity of a “pediatric care”
The child must be operated by the pediatric surgeon
The dedicated and skilled surgical nursing team: to be recovered
The “functional pediatric surgical model”: establish in General Hospitals
Spoke centers: surgery ensured by a pediatric team

To achieve this fundamental objective, it is necessary to resort to some urgent measures. First, it is a formalization of the concept that pediatric patients should be operated on by pediatric surgeons. Second, the development of a collaborative relationship with other satellite pediatric surgical specialties (ENT, maxillofacial, orthopedics, neurosurgery, etc.) is urgently required to identify pediatric paths dedicated to children to give back the child the dignity of care that the COVID-19 has subtracted. The COVID-19 pandemic has increased the already high number of children admitted to adult wards while children should be admitted only in a dedicated pediatric environment. Considering the high number of pediatric patients hospitalized in adult wards, the time has come to reiterate the concept of pediatric age limits valid for all Italian hospitals. It must be recalled that the first article of the “Convention on the Rights of the child” defines a child as “every human being below the age of 18 years”.^1^ Furthermore, the obligation to treat children only in the pediatric environment is proposed by force, as also confirmed by the charter of the rights of the child. The implementation of pediatric surgery in spoke centers can promote the hub-spoke network and help limit the hospitalization of children in adult wards. This project must be supported by a pediatric anesthesiology team that recruits children over the age of 5 years in spoke centers. Accordingly, an anesthesiologist at spoke centers needs dedicated training as required by the regulation on hospital care standards.

On the other hand, there is a complete lack of a project involving the whole of Italy on the number of centers necessary to cover the needs of the population. There is formal and substantial disarray because the regions work without general coordination that should give behavioral guidelines to the identification and authorization to open centers that should respond to clear and precise criteria. Pediatric surgery centers must be identified based on the needs of the population and the territory and divided into treatment areas (who is authorized to do what). Having, for example, 150 cases of kidney tumors operated in 56 different centers in Italy does not allow to develop and maintain the needed skills neither for surgeons nor for the whole structures.

Table 5 summarizes the administrative proposals to limit pediatric surgical patients treated in general hospitals.

**Table 5:** Criticalities and proposals to limit admissions of children in adult wards.

**Criticalities**	**Intervention**
N of Pediatric Surgery Centers in Italy	National vision and coordination needed
Pediatric age limits in the wards	A national uniformity is needed
Hospitalization of children in adult wards	A national provision should limit and put an end
DRG not appropriate in pediatric age	Updated DRGs according to the “complexity” of pediatric surgery management

Needs are changing rapidly. Day surgery function is increasing in all units, which constitutes a very important percentage of pediatric surgical activity. Alongside this, outpatient surgery, which has helped to reduce hospitalization, especially in chronic patients, requires space and time and dedicated staff, as well as a reorganization of nursing activities. Day surgery, outpatient surgery, and day hospital, all require investment and adaptation as they offer a quick service to the territory by reducing hospitalization, related costs, and inconveniences for the family. Such important projects of quality childcare and cost reduction are not supported by national health projects for the requalification of personnel, spaces, and related regulations.

Surgical technology is advancing rapidly. In recent years, we have witnessed technological innovations in pediatric surgery with the integration of minimally invasive, robotic, and virtual reality surgery even in young patients. The response that pediatric surgery has given to the reorganization required by the pandemic has reinforced the need to implement telemedicine in clinical practice, especially aimed at chronic and frail patients. According to data reported by the activity of social and health professionals, the use of telemedicine increased significantly during the COVID-19 pandemic, also in some pediatric surgery settings (15). Pediatric surgery centers could be placed to expand and regulate innovations, such as images, in real-time. The role of the pediatric nursing staff nurses dedicate to such technologies need to be included in a national health project that protects the figure of the pediatric nurse with the same rights as the professional nurse.

Finally, there is a lack of specialists due to past mistakes and this creates enormous discomfort. In the current attributions to post-graduate training schools (therefore with a 5-year vision), old evaluations have been used that do not meet the criteria of receptive as well as didactic capacity. Regarding surgeons in training, the legislation is very restrictive in Italy. Absurdly, a recent graduate could, in theory, be hired in cardiac surgery and operate as the first heart operator, but a surgical trainee, for example, in pediatric surgery cannot do tutoring on call. Table 6 shows proposals to improve training in pediatric surgery in Italy. It is necessary to give an operational role to post-graduate training schools in terms of rigorous accreditation criteria, commonality of programs, interchangeability to acquire knowledge and skills also from comparison with other centers.

**Table 6:** Training of new pediatric surgery specialists.

Organization of post-graduate training schools: commonality of programs among all schools
Rigorous accreditation criteria, commonality of programs, interchangeability to acquire knowledge and skills also from comparison with other Centers
Give an operational role—now very forced by legislation—to doctors in training

The legislation is also lacking from the point of view of the responsibility of health professionals operating in the national pediatric field. Italian legislation is not the same as that enjoyed by other professionals (magistrates, notaries, lawyers, etc.), and in most cases, pediatric expertise is supported by adult professionals who have no experience in pediatric surgical pathology.

New protocols of pediatric care could be taken into consideration as possible scenarios for each hospital. Surgical teams are asked to adhere to national and regional guidelines, to evidence their expertise, and the availability of resources dedicated to children.

## Conclusion

The COVID-19 pandemic has upset the assistance in the pediatric area. As a consequence, the pediatric surgical organization in general hospitals and pediatric hospitals has been completely subverted. The COVID-19 infection is still spreading and children are seriously penalized both for surgical treatment as well as for the diagnostic component. In most centers, the pediatric surgical teams are the adult ones and the specificity of training the pediatric operating nursing is in danger of survival. Moreover, the pandemic has removed the “emotional” aspects of the admitted child has lost its central position in patients' management and the risk is real that in general hospitals the child will once again be considered a small adult. Given all these criticalities, all efforts should be done to define guidelines on the management of surgical children, both in pandemic times and in normal ones to ensure the specificity of treatment and give back the child the dignity of care that the COVID-19 has subtracted.

## Author Contributions

FM and GP contributed to the conception and design of the study and wrote the first draft of the manuscript. All authors contributed to the article and approved the submitted version.

## Conflict of Interest

The authors declare that the research was conducted in the absence of any commercial or financial relationships that could be construed as a potential conflict of interest. The reviewer FG declared a shared affiliation with the authors FM to the handling editor at the time of review.

## Publisher's Note

All claims expressed in this article are solely those of the authors and do not necessarily represent those of their affiliated organizations, or those of the publisher, the editors and the reviewers. Any product that may be evaluated in this article, or claim that may be made by its manufacturer, is not guaranteed or endorsed by the publisher.

## Footnotes

^1^https://www.ohchr.org/en/professionalinterest/pages/crc.aspx (accessed November 25, 2021).

## Appendix

Predefined questions to be used as a canvas:

1. Pre-pandemic criticalities: starting with the criticalities that each pediatric society had expressed in the FIARPED white paper.
2. Pediatric care during the pandemic and recovery describing:
  - What happened compared to what we had assumed?
  - How we reorganized?
  - What if the reorganization has remained in our daily business now that we are emerging from the pandemic?
3. Current criticalities: What are the criticalities that your specialty is experiencing in this moment of change?
4. The future care model of our specialty.
